# Cellular senescence: a key therapeutic target in aging and diseases

**DOI:** 10.1172/JCI158450

**Published:** 2022-08-01

**Authors:** Lei Zhang, Louise E. Pitcher, Matthew J. Yousefzadeh, Laura J. Niedernhofer, Paul D. Robbins, Yi Zhu

**Affiliations:** 1 Institute on the Biology of Aging and Metabolism and the Department of Biochemistry, Molecular Biology and Biophysics, University of Minnesota, Minneapolis, Minnesota, USA.; 2 Robert and Arlene Kogod Center on Aging, and; 3 Department of Physiology and Biomedical Engineering, Mayo Clinic, Rochester, Minnesota, USA.

## Abstract

Cellular senescence is a hallmark of aging defined by stable exit from the cell cycle in response to cellular damage and stress. Senescent cells (SnCs) can develop a characteristic pathogenic senescence-associated secretory phenotype (SASP) that drives secondary senescence and disrupts tissue homeostasis, resulting in loss of tissue repair and regeneration. The use of transgenic mouse models in which SnCs can be genetically ablated has established a key role for SnCs in driving aging and age-related disease. Importantly, senotherapeutics have been developed to pharmacologically eliminate SnCs, termed senolytics, or suppress the SASP and other markers of senescence, termed senomorphics. Based on extensive preclinical studies as well as small clinical trials demonstrating the benefits of senotherapeutics, multiple clinical trials are under way. This Review discusses the role of SnCs in aging and age-related diseases, strategies to target SnCs, approaches to discover and develop senotherapeutics, and preclinical and clinical advances of senolytics.

## Introduction

**Figure 1:**
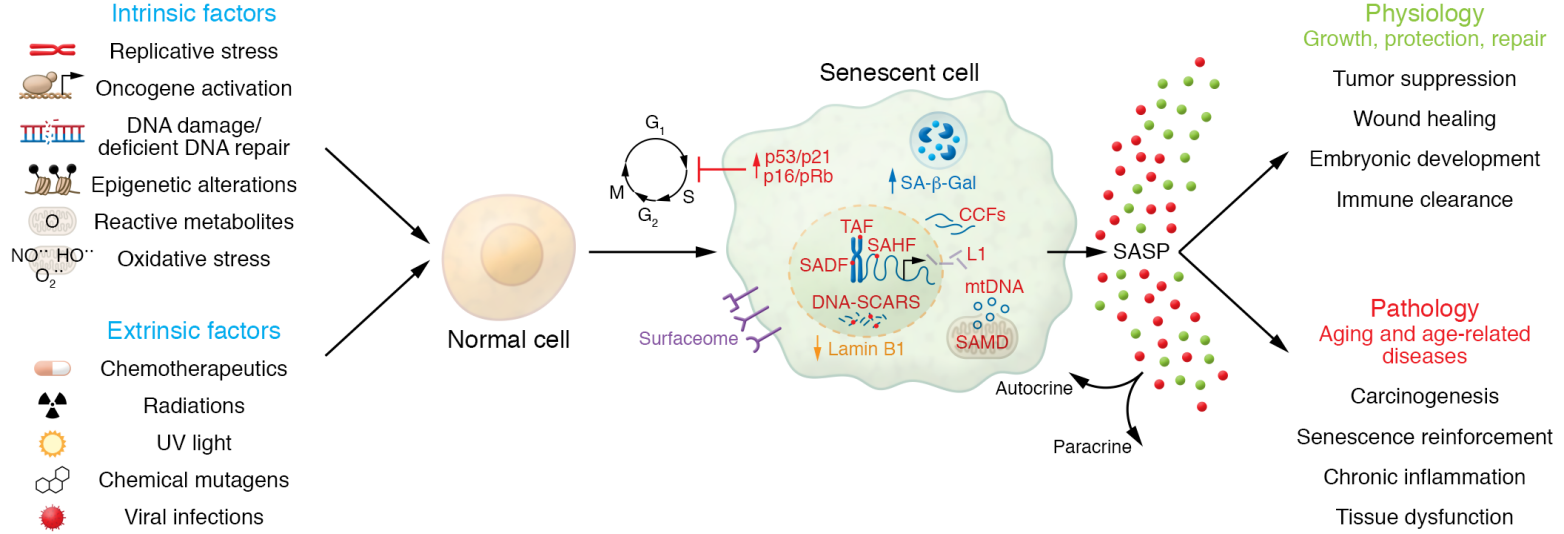
Diverse stress stimuli can induce cellular senescence and lead to generation of senescent cells, which play pleiotropic roles in both physiology and pathology. CCF, cytoplasmic chromatin fragment; DNA-SCARS, DNA segments with chromatin alterations reinforcing senescence; mtDNA, mitochondrial DNA; SADF, senescence-associated DNA damage foci; SAHF, senescence-associated heterochromatin foci; SAMD, senescence-associated mitochondrial dysfunction; SASP, senescence-associated secretory phenotype; TAF, telomere-associated foci.

Aging is a complex process driven, at least in part, by hallmarks of aging, including cellular senescence, genomic instability, telomere attrition, epigenetic alterations, loss of proteostasis, deregulated nutrient sensing, mitochondrial dysfunction, stem cell exhaustion, and altered intercellular communication (1, 2). Of these hallmarks, cellular senescence has been directly implicated as a key driver of aging and age-related diseases. Senescent cells (SnCs) are characterized by stable exit from the cell cycle and loss of proliferative capacity, even in the presence of mitogenic stimuli (3). The phenomenon of cellular senescence was first described by Leonard Hayflick and Paul Moorhead in 1961 using diploid fibroblast cell lines, which failed to further divide after 40–60 population doublings (4). In addition to replicative senescence caused by telomeric erosion and induction of a DNA damage response, cellular senescence can be induced by other stressors, including but not limited to epigenetic changes, genomic instability, mitochondrial dysfunction, reactive metabolites, oxidative stress, inactivation of certain tumor suppressor genes, oncogenic- and therapy-induced stress, and viral infections (Figure 1) (3, 5–12).

SnCs exhibit molecular features (e.g., expression of senescence markers) and morphological features (e.g., enlarged, flattened appearance) that make them distinguishable from normal cells (Figure 1) (13). Lysosomal hydrolase activity at pH 6.0 resulting from enhanced lysosomal biogenesis, termed senescence-associated β-galactosidase (SA-β-gal), and accumulation of cytoplasmic granules in the lysosomes leading to an enlarged hypertrophic morphology are prominent phenotypes of SnCs (3, 14). The cellular senescence program is initiated by the p16^INK4a^/Rb and/or p53/p21^CIP1^ tumor suppressor pathways. p16^INK4a^, p21^CIP1^, and p53 are cyclin-dependent kinase inhibitors and a tumor suppressor that act in a coordinated manner and/or independently to arrest cell cycles in G_1_ (15–17). Expression of *p16^INK4a^*, known to increase in mammalian tissue with age, is a prominent marker of cellular senescence (18–20). For example, *p16^INK4a^* expression in CD3^+^ human peripheral blood T lymphocytes is a robust marker of chronological and biological age (21). In addition to increased *p16^INK4a^* and *p21^CIP1^* expression, SnCs have reduced lamin B1, reflecting disruption of the nuclear lamina observed during senescence (22), which is emerging as another hallmark of SnCs (23). Epigenetic changes in SnCs create an environment that is permissive to the deregulation of transposable elements like LINE-1, whose increased expression is observed in late senescence (24, 25). Furthermore, SnCs are characterized by the presence of DNA damage–associated features such as DNA segments with chromatin alterations reinforcing senescence (DNA-SCARS) and senescence-associated heterochromatin foci (26, 27). These alterations in the nuclear architecture of the genome are potentially involved in determining the cell fate decision for cellular senescence (28).

Although SnCs are growth arrested in the cell cycle, they are still metabolically active (8). Many SnCs secrete a wide spectrum of bioactive factors, including inflammatory cytokines, chemokines, growth factors, matrix metalloproteinases, lipids, nucleotides, extracellular vesicles, and soluble factors, termed the senescence-associated secretory phenotype (SASP) (29). In addition, the release of DNA from the nucleus, termed cytoplasmic chromatin fragments, resulting from nuclear-cytoplasmic blebbing, mitochondrial DNA, NF-κB signaling, and the C/EBPβ transcription cofactor is an important factor in priming the SASP (30–32).

## Senescent cells in aging and age-related diseases

Cellular senescence is thought to have evolved as an antitumor mechanism where the SASP induced by oncogene-induced senescence recruits immune cells to facilitate SnC removal (33, 34). SnCs are heterogeneous in nature and emerge throughout life due to various stimuli. In addition to heterogeneity, SnCs are also pleiotropic in function. SnCs play an essential role in multiple physiological processes, including embryogenesis, cellular reprogramming, tissue regeneration, wound healing, immunosurveillance, and tumor suppression (35–41). However, SnCs can also contribute to the pathology of many chronic diseases, including diabetes, cancer, osteoarthritis, and Alzheimer’s disease (Figure 1) (42, 43). SnCs accumulate with age in most tissues, and SASP factors can act in both proximal and distal fashions to induce secondary senescence, thus propagating and enhancing the SnC burden (3, 29). The SASP also serves to sustain and enhance inflammaging, whereby enhanced chronic, low-grade systemic inflammation occurs in the absence of pathogenic processes (44, 45).

The use of *p16^Ink4a^*-high senescent cell reporter mice (*p16^LUC^* and *p16*-CreERT2-tdTomato mice) revealed that *p16^Ink4a^*-expressing SnCs progressively increased with age and drove aging and cancer processes in mice (18, 46, 47). In several mouse models of accelerated aging, *Ercc1^–/Δ^* and *Bub1b^H/H^* hypomorphic mice develop genomic instability with accelerated accumulation of SnCs, resulting in premature aging symptoms including shortened lifespan and increased histopathological lesions in various organs (48–50). Use of a transgenic *p16^Ink4a^*-expressing SnC removal system in mice, termed INK-ATTAC, provided further evidence of a key role for SnCs in aging and disease. This model enables selective clearance of SnCs using the promoter of *p16^Ink4a^* combined with an FKBP–caspase-8 suicide transgene that induces apoptosis of *p16^Ink4a^*-high SnCs through targeted activation of caspase-8 (51) with an FKBP dimerizer. This genetic clearance strategy was first applied in *Bub1b^H/H^*-progeroid mice, in which it blunted age-related pathologies including sarcopenia, cataracts, and lipodystrophy (51). The INK-ATTAC model also was used to demonstrate the beneficial consequences of SnC clearance in naturally aged mice, where it improved healthspan, extended median lifespan, and delayed tumorigenesis.

Cellular senescence not only contributes to aging but also plays a causal role in numerous age-related diseases. SnC accumulation frequently occurs at pathogenic sites in many major age-related chronic diseases, including Alzheimer’s and cardiovascular diseases, osteoporosis, diabetes, renal disease, and liver cirrhosis (52–56). Notably, transplanting a small number of SnCs into young healthy animals recapitulates age-related impaired physical functions (57, 58). This supports the threshold hypothesis, which proposes that once the SnC burden increases beyond sustainability in a tissue, it activates age-related pathological changes and eventually results in disease. Genetic clearance of *p16^Ink4a^*-high SnCs in the INK-ATTAC mouse models has demonstrated the benefit of SnC clearance in the prevention or alleviation of diseases including osteoporosis, frailty, atherosclerosis, hepatic steatosis, osteoarthritis, idiopathic pulmonary fibrosis, obesity-induced anxiety, tau-mediated neurodegenerative disease, and type 2 diabetes mellitus/metabolic dysfunction (51, 59–62). Consistently, studies using the transgenic p16-3MR mouse model, expressing luciferase and red fluorescent protein (RFP) reporters and herpes simplex virus-1 thymidine kinase, which converts ganciclovir into an apoptosis inducer, showed that ganciclovir-induced genetic depletion of *p16^Ink4a^*-expressing SnCs alleviates multiple age-related dysfunctions (60, 63, 64). Despite the use of a transgenic *p16^Ink4a^*-expressing SnC removal system in mice to study SnCs in aging and age-related diseases, the mouse model relies on the expression of *p16^Ink4^* alone. This is potentially problematic as not all *p16^Ink4a^*-expressing cells are detrimental, and some are physiologically beneficial (65). Also, there are SnCs that are not *p16^Ink4a^* positive. Mouse models using more precise senescence markers need to be developed in the future to clarify the controversial findings.

The deleterious effects of SnCs in aging and many age-related diseases are likely mediated by increased SASP expression (29, 66). SASP factors, such as TGF-β family members, VEGF, and chemokines, are known to accelerate senescence accumulation by spreading senescence to neighboring cells (67, 68). The SASP crosstalk with immune cells, including NK cells, macrophages, and T cells, exacerbates both local and systemic inflammation (69). Proteases and growth factors in the SASP are known to disrupt tissue microenvironments and promote cancer metastasis (29). Fibrogenic factors and tissue remodeling factors in the SASP contribute to fibrosis in multiple tissues, including skin, liver, kidney, lung, cardiac tissue, pancreas, and skeletal muscle (70). Since the SASP contributes to diseases by disrupting tissue homeostasis, suppressing the SASP is an alternative strategy to alleviate the detrimental effects of SnCs in multiple studies. For example, inhibiting the SASP reduces inflammation, restores insulin sensitivity, blunts osteoporosis, and improves physical functions in aged mice (71–74). Abolishing NF-κB–dependent SASP delayed the onset of progeroid symptoms and extended healthspan in *Ercc1^–/Δ^* mice (75). mTOR signaling also modulates the SASP, and rapamycin, a selective inhibitor of mTOR complex 1 (mTORC1), strongly impairs the SASP, reduces inflammation, and extends healthspan and lifespan (76–79).

Collectively, the accumulation of SnCs in tissues with age, along with the detrimental effects of the SASP, is a potent driver of aging and age-related pathologies, shortening both healthspan and lifespan. Importantly, removal of SnCs, or suppression of the SASP, can alleviate or delay multiple chronic age-related conditions, demonstrating the therapeutic potential of targeting SnCs (58, 80, 81).

## Strategies to target senescent cells therapeutically

**Figure 2:**
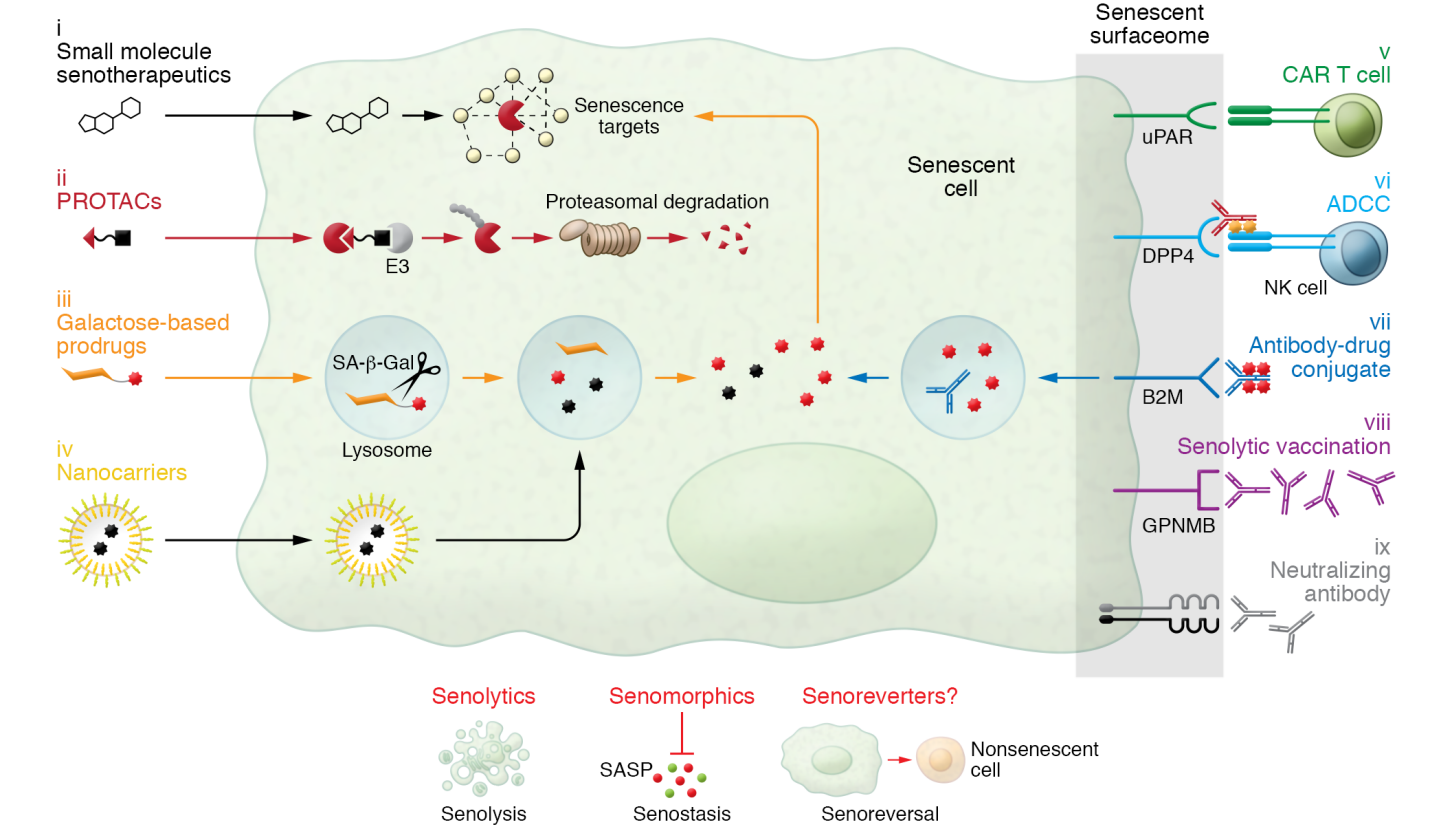
Current strategies to target senescent cells.

Due to the therapeutic potential of reducing the SnC burden to extend healthspan and delay the onset of age-related diseases, there is growing interest in developing senotherapeutics that incorporate multidisciplinary technologies from diverse fields such as biology, chemistry, nanotechnology, and immunology (82–85). Both intracellular senescence-associated pathways and extracellular membrane proteins upregulated on the surface of SnCs, termed the senescent surfaceome (86, 87), can be exploited for therapeutic as well as diagnostic purposes (Figure 2). Current senotherapeutic strategies targeting SnCs include conventional senotherapeutics, prodrugs, protein degraders, nanocarriers, and immunotherapies.

### Senolytics.

SnCs upregulate distinct antiapoptotic pathways (SCAPs) for survival, which can serve as molecular targets for pharmacological interventions to promote senolysis. Several SCAPs and their key proteins have been identified as senolytic targets for drug development. The feasibility of targeting SnCs was first demonstrated using the senolytic combination of dasatinib plus quercetin (D+Q) (88). Subsequently, many other senolytics have been reported, including inhibitors of the antiapoptotic BCL-2 family proteins (e.g., navitoclax/ABT-263, ABT-737), HSP90 inhibitors, USP7 inhibitors, p53 modulators (e.g., inhibitors of FOXO4-p53 or MDM2-p53 interactions), Na^+^/K^+^-ATPase inhibitors (e.g., cardiac glycosides), and others (84, 85). In addition, certain natural products, such as fisetin, piperlongumine, and curcumin, also have been identified as senolytics, though their exact mechanisms of action are unclear (84, 85). To date, the two most studied senolytics are D+Q and fisetin, both of which have entered different clinical trials for treatment of age-related diseases (Table 1).

### Senomorphics.

Compounds that reduce the detrimental effects of the SASP or suppress senescence without inducing SnC death are termed senomorphics, also known as senostatics. Most senomorphics act by interfering with transcriptional regulators of the SASP, such as inhibitors of ATM, p38 MAPK, JAK/STAT, and the NF-κB and mTOR pathways (84, 85). One possible limitation of senomorphics is that they likely require continuous administration, as opposed to senolytics, which require only intermittent administration because of their hit-and-run mechanism (89). Notably, depending on cell types and treatment concentrations, some compounds have been shown to exhibit both senolytic and senomorphic effects. For instance, procyanidin C1, a polyphenolic flavonoid isolated from grape seed extract, is senomorphic at low concentrations but senolytic at higher concentrations (90).

### Senoreverters.

Although cellular senescence is generally thought to be an irreversible cell fate, recent studies suggest that senescence in certain cell types is a dynamic process that can be reverted to allow SnCs to reenter the cell cycle (91, 92). For example, the suppression of NF-κB and mTOR signaling and inhibition of 3-phosphoinositide–dependent protein kinase 1 (PDK1) in senescent human dermal fibroblasts removed senescence hallmarks, and converted the cells from a senescent to a quiescent state, resulting in restored skin regeneration capacity (93). Also, a specific six-factor gene cocktail reversed cellular senescence of senescent and centenarian fibroblasts and reprogrammed them into pluripotent stem cells (94). Thus senoreverters may provide a third therapeutic approach to target SnCs (Figure 2) (95). However, there is also evidence that therapy-induced SnCs can escape the senescence state and acquire stemness features as well as more aggressive tumor growth potential through activated Wnt signaling (96). Given that cellular senescence is a protective mechanism that suppresses tumorigenesis and metastasis (97), testing the safety of senoreverters will be extremely important.

### Galactose-based prodrugs.

A common characteristic of SnCs is increased lysosomal SA-β-gal activity, which hydrolyzes the β-glycosidic bond formed between a galactose and its organic moiety. This enzymatic activity can be exploited to design galactose-based prodrugs by covalently attaching galactose or acetyl galactose groups to a cytotoxic molecule. The fused galactoside prodrugs are processed preferentially in SnCs after cellular uptake, resulting in the release of active cytotoxic drugs and selective killing of SnCs. The feasibility of this strategy has been demonstrated by several galactose-based prodrugs, notably SSK1, prodrug A (JHB75B), and Nav-Gal (98–100). Interestingly, the cytotoxic moieties of these prodrugs are all chemotherapeutic reagents, such as gemcitabine (98), duocarmycin (99), and 5-fluorouracil and navitoclax (100), respectively. In theory, this prodrug strategy increases the selective killing of SnCs over non-senescent normal and proliferative cells. For example, Nav-Gal has reduced platelet toxicity in comparison with the parent drug navitoclax (100).

### Proteolysis-targeting chimera (PROTAC).

Proteolysis-targeting chimeras (PROTACs) are an innovative technology to induce degradation of a protein of interest (POI) (101). PROTACs are heterobifunctional molecules composed of three elements: a ligand that binds to a target POI, an E3 ligase recruiting ligand, and a flexible linker between the two ligands. Thus, a PROTAC can form a stable ternary complex with a POI and E3 ligase (102), resulting in subsequent ubiquitination and proteasomal degradation of the POI. PROTACs have several advantages, such as increased potency, higher selectivity, prolonged activity, and reduced toxicity, which make them an attractive strategy for developing senotherapeutics (103). Several PROTAC-based senolytics have already been developed. For instance, PZ15227 was generated by tethering of the senolytic drug navitoclax (ABT-263) to a cereblon (CRBN) E3 ligand that is expressed minimally in normal platelets (104). PZ15227 showed increased efficacy and potency compared with navitoclax in clearing SnCs while causing less cytotoxicity to platelets than navitoclax (104). Another example is the attachment of the BET inhibitor OTX015 to the E3 ligase binder pomalidomide to create a novel bifunctional PROTAC, ARV825, that acts as a BET family protein degrader (105). ARV825 promoted BRD4 degradation and displayed robust senolytic activity in SnCs even at nanomolar concentrations and was able to eliminate SnCs in mouse models (105). Predictably, other SCAP targets also can be exploited to develop more PROTAC senotherapeutics. However, PROTACs usually have a higher molecular weight than traditional small molecules, and therefore they may have less optimal pharmacokinetic properties and may not be suitable for oral delivery. Another caveat to be considered in cytotoxicity studies of bivalent PROTACs is that they may exhibit reduced degradation at high concentrations, a phenomenon referred to as the hook effect (101, 102). It is also worth mentioning that resistance to PROTAC effects may arise if mutations in the POI or the core components of E3 ligase complexes occur (106, 107).

### Nanocarriers.

Owing to its modifiable physiochemical properties, nanotechnology allows controlled delivery and release of various payloads to targeted cells, making it an enabling technology for detection, diagnosis, and drug delivery in cancer treatment. Many nanomaterials, particularly nanoparticles (NPs), have been tailor-made as nanocarriers to target SnCs for senescence detection and therapeutic interventions (108). The large surface area of NPs can be covalently modified with different functionalities such as peptides, antibodies, or nucleic acids. Additionally, the characteristic SA-β-gal can be used to produce NPs conjugated with galacto-oligosaccharides for preferential delivery into SnCs. For example, doxorubicin and navitoclax were encapsulated in NPs as payloads to generate senolytic nanoparticles GalNP(dox) and GalNP(nav) (109). After cellular uptake via endocytosis, fusion with lysosomal vesicles, and hydrolysis of the galacto-oligosaccharide coat by SA-β-gal, these NPs released their free cytotoxic cargoes to selectively kill SnCs while sparing normal healthy cells (109). Modification with β-galactose groups on self-assembling peptides was also reported to allow their selective cellular uptake by SnCs followed by specific SA-β-gal cleavage, resulting in an enzyme-instructed self-assembly process that forms intracellular nanofibers and hydrogels, eventually triggering SnC death by activating their apoptotic pathways (110). Another layer of specific delivery of NPs to SnCs can be achieved by combining lactose encapsulation with the senescent surfaceome. For example, the senomorphic drug rapamycin was loaded in lactose-wrapped calcium carbonate NPs that were additionally conjugated with a monoclonal antibody against CD9, overexpressed on some SnCs (111). Upon intracellular drug delivery in old human dermal fibroblasts, the dual-functional CD9-Lac/CaCO_3_/Rapa NPs exhibited high uptake by SnCs via surface recognition and anti-senescence effects (111). Molecularly imprinted nanopolymers (nanoMIPs) (112–114) were also generated to target SnCs based on β_2_-microglobulin (B2M) epitope, another senescent surfaceome protein (115). B2M nanoMIPs loaded with dasatinib demonstrated selective killing of SnCs over proliferating cells and improved potency over dasatinib alone, minimizing the off-target toxicity of dasatinib (116). Other kinds of NPs have also been reported, including molybdenum disulfide nanoparticles (117), zinc oxide nanoparticles (118), and quercetin surface-functionalized magnetite Fe_3_O_4_ nanoparticles (119). However, they were not functionalized to preferentially target SnCs.

### Immunotherapy based on the senescent cell surfaceome.

The immune system plays an important role in clearing SnCs. Under physiological conditions, SnCs can stimulate both innate and adaptive immune responses by secreting SASP factors or elevating particular surface antigens in order to recruit immune cells, such as T cells, macrophages, NK cells, and neutrophils, for their clearance (120–122). However, aging of the immune system, termed immunosenescence, results in declined immunosurveillance. SnCs also may develop immune suppression programs that enable them to resist immune clearance. These may be attributable in part to SnC accumulation in tissues and associated tissue dysfunctions. Therefore, immunotherapy based on stimulating the ability of immune cells to target SnCs represents an alternative senotherapeutic strategy. Such immunotherapies usually take advantage of senescent surfaceome proteins composed of antigens and receptors preferentially upregulated on the surface membrane of SnCs, including B2M, urokinase-type plasminogen activator receptor (uPAR), dipeptidyl peptidase 4 (DPP4), glycoprotein nonmetastatic melanoma protein B (GPNMB), CD9 receptor, NOTCH receptors, and others (86, 87). Chimeric antigen receptor (CAR) T cells are an anticancer therapy involving the genetic re-engineering of T cells to produce an artificial receptor antigen that helps them recognize and destroy targeted cancer cells. In a recent study, CAR T cells were redirected to recognize the senescent surfaceome protein uPAR to preferentially eradicate SnCs in different in vitro and in vivo models of senescence (123). It is possible that CAR NK cells or CAR macrophages could be developed in a similar manner to enhance cytotoxic activity against SnCs. Antibody-dependent cellular cytotoxicity (ADCC), also known as antibody-dependent cell-mediated cytotoxicity, is another type of immunotherapy in which antibodies are used to guide immune cells toward cytotoxic clearance of target cells. A DPP4-based ADCC approach was developed by labeling of DPP4-bearing SnCs with an anti-DPP4 antibody to guide NK cells to selectively clear the DPP4-positive SnCs (124). Antibody-drug conjugates (ADCs) are monoclonal antibodies attached to cytotoxic drugs that have been successfully used for the treatment of many cancers. The first senolytic ADC was designed by conjugation of a B2M IgG1 monoclonal antibody with duocarmycin, an irreversible DNA alkylating agent (125). This B2M-duocarmycin ADC recognized and bound to the extracellular epitope B2M on the surface of SnCs. After internalization via endolysosomal trafficking, it can be cleaved by cathepsin B to release duocarmycin, causing selective cell death of SnCs (125). Senolytic vaccination could be another option for senolytic immunotherapy and requires only one or a few treatments. For instance, immunization of progeroid mice with a senolytic GPNMB vaccine developed using GPNMB-derived peptides resulted in reduced GPNMB-positive SnCs, improved pathological phenotypes associated with aging, and extended lifespan (126). Neutralizing antibodies can also be used to target specific SASP components (e.g., IL-1β, IL-6, and IL-8) of SnCs or block their upstream surface receptors for senescence suppression. Some antibodies have been approved for treatment of immune disorders, for example, the anti–IL-6 antibody siltuximab (127); tocilizumab, targeting the IL-6 receptor (128); and the anti–IL-1β antibody canakinumab (129). However, their effects in the context of senescence are yet to be determined.

## Approaches to discover and develop novel senotherapeutics

**Figure 3:**
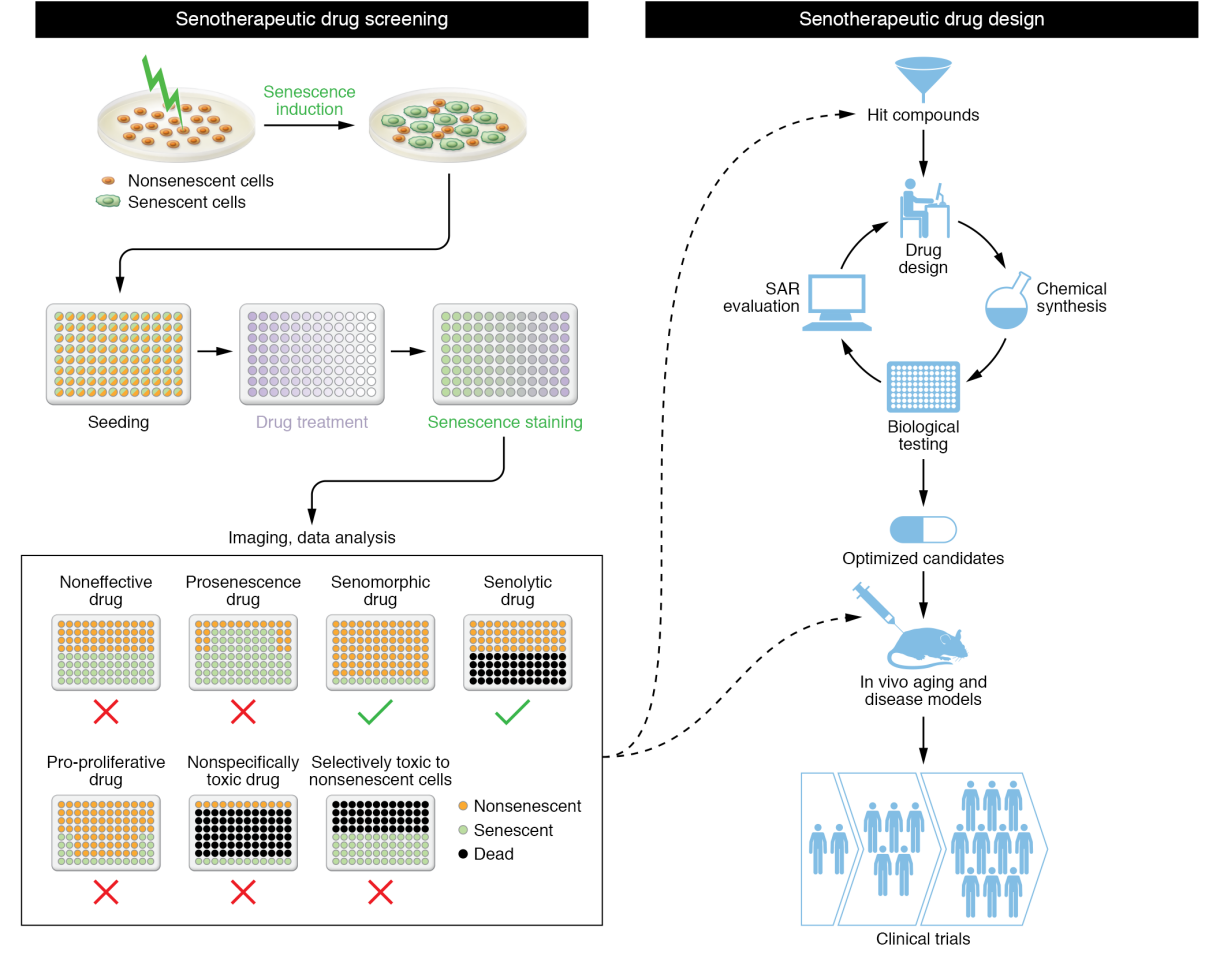
Drug screening and drug design can facilitate the discovery and development of senotherapeutics to treat aging and age-related diseases.

As discussed above, numerous strategies have been developed to target the pathological effects of SnCs, including induction of SnC death (senolysis), suppression of the detrimental effects of the SASP (senostasis), and possible rejuvenation of senescence status (senoreversal) (Figure 2). As such, small-molecule senotherapeutics, especially senolytics, hold great translational potential and have attracted considerable interest in academia and industry. To date, most senotherapeutics have been discovered through bioinformatics approaches and/or focused library screening (84, 85). However, modern advances in drug screening and drug design can facilitate the discovery and development of novel senotherapeutics (Figure 3).

### Drug screening.

The target of senotherapeutics is SnCs; therefore, in vitro SnC-based drug screening serves as a major source of senotherapeutic drug discovery. Given the heterogeneity of SnCs, different cell types can be cultured and induced to senescence under different stress conditions to recapitulate the heterogeneity. Upon drug treatment, the senotherapeutic effects on SnCs can be detected and evaluated via cell viability or different senoprobes such as SA-β-gal–based chemogenic or fluorogenic dyes (130, 131). Both senolytics and senomorphics could be identified from such phenotypic drug screening. Complementary to the phenotypic cell-based screening of chemicals, genetic screening of vulnerabilities of SnCs can provide new senescence targets for drug discovery. For example, genome-wide CRISPR/Cas9–based screening has been applied to identify genes that potentially regulate cellular senescence, such as *SMARCB1*, coagulation factor IX (*F9*), and *KAT7* (132–134). With a deeper understanding of senescence biology and the discovery of more senescence targets, structure-based virtual screening should be feasible using certain key proteins and pathways regulating senescence. Alternatively, computer-aided drug design (CADD), artificial intelligence (AI), and machine learning (ML) technologies also can be used to complement senotherapeutic screening. The feasibility has been demonstrated in a recent study in which a deep learning–based senescence scoring system by morphology (Deep-SeSMo) based on pretrained convolutional neural networks could identify SnCs and evaluate senescence (135).

### Drug design.

Drug screening is an effective method for discovering new senotherapeutics, but it relies heavily on existing chemical libraries and thus rarely generates new chemical entities. In contrast, rational drug design is well suited to the discovery of specific molecular target–based senotherapeutics with novel chemical structures (104). Furthermore, it can be used to optimize any hits from senescence drug screening as well as reported senotherapeutics in order to improve potency and achieve desirable drug-like properties including bioavailability and pharmacokinetics by multiple rounds of structure-activity relationship studies (136). In addition to traditional medicinal chemistry, CADD, AI, and ML also can be applied to aid the design and development of new senotherapeutics.

## Senolytics in preclinical animal models

There is a rapidly growing body of evidence of the effectiveness of senolytics in vivo in animal models of diseases and aging (137). Senolytics can be evaluated for their beneficial effects on both healthspan and lifespan using longitudinal measurements (e.g., body composition, echocardiography, grip strength and rotarod, glucose and insulin tolerance tests), in addition to commonly used frailty scoring systems (58, 138–140). The use of the geropathology grading platform to assess aging-specific lesions in tissues is an additional way to evaluate healthspan in a postmortem manner (141, 142). While testing in naturally aged mice is critical to assessing senolytic efficacy, accelerated aging models of Hutchinson-Gilford progeria syndrome or XFE progeria can serve as a time- and cost-efficient alternative to test senolytics in vivo (143, 144). Testing senolytics in animal models of senescence-associated diseases (e.g., cardiovascular disease, cognitive impairment, diabetes mellitus and metabolic syndrome, idiopathic pulmonary fibrosis, osteoarthritis, pathogenic infection, therapy-induced frailty) can provide useful preclinical data to assess clinical indications of identified senolytics (58, 61, 145–148). Indeed, senolytics have been shown to alleviate many conditions in many preclinical mouse models of diseases (84, 85, 89, 149), as we summarize below.

The first discovered senolytic, the combination of D+Q, is currently the most studied senolytic in preclinical animal models. Treatment with D+Q increased the lifespan in naturally aged mice, extended healthspan in progeroid and naturally aged mice, and ameliorated premature frailty and morbidities caused by radiation or chemo-drugs in mouse models mimicking cancer survivors (58, 88, 150–154). Interventions using D+Q alleviated multiple senescence-associated tissue dysfunctions, including lung, fat, muscle, kidney, liver, heart, bone, vascular, and brain, in various age-related chronic disease models and organs (88, 146, 147, 155–162). The therapeutic effects of D+Q in multiple diseases are summarized in Table 2. The heterogeneity and complexity of SnCs suggest that seeking universal senolytics across all cell types and tissues might not be realistic. This reasoning also accounts for observations that D+Q does not induce apoptosis in certain types of SnCs (163, 164).

Another senolytic agent, the natural flavonoid compound fisetin, showed efficacy in multiple senescence-associated disorders in mice. The senolytic action of fisetin was first tested in both naturally aged and progeroid mouse models, where its treatment improved healthspan and extended median and maximum lifespan (165). In addition, fisetin enhanced the immune response of old mice exposed to pathogens, including the mouse hepatitis virus, a β-coronavirus, and reduced mortality (11). This result suggests that targeting SnCs could prevent severe symptoms and mortality in the elderly. In the tau transgenic (rTg4510) mouse model of Alzheimer’s disease (AD), fisetin treatment greatly improved both cognitive and physical functions (151).

Navitoclax (ABT-263), a compound targeting one type of SCAPs, the BCL-2 family of proteins, is another extensively studied senolytic compound showing comparable outcomes to D+Q and fisetin in multiple mouse models (Table 2) (60, 61, 161, 162, 166–170). Although navitoclax showed diverse benefits in many preclinical tests, its translational potential is still limited by its platelet toxicity (171, 172).

The senolytic FOXO4-DRI peptide, designed to target another important node on a SCAP, the FOXO4-p53 complex, ameliorated premature aging pathologies caused by doxorubicin-induced chemotoxicity (163). Recently a newer version of FOXO4-DRI, the ES2 peptide, has been developed using a precise molecular modeling approach showing improved therapeutic effects in comparison with conventional chemotherapeutics in a preclinical model of melanoma (173, 174).

However, it is important to note that despite the enormous therapeutic potential of senotherapeutics shown in various preclinical tests, the efficacy and potential adverse effects should be formally assessed in human clinical trials.

## Senolytics in clinical studies

Preclinical studies demonstrated that the accumulation of SnCs with age drives age-related diseases and pharmacologically clearing SnCs can alleviate pathologies in preclinical models of age-associated disorders including cancer, cardiovascular disease, and neurological, liver, kidney, musculoskeletal, lung, eye, hematological, metabolic, and skin diseases, even at a late life stage (89). These extensive preclinical studies were used to support the rapid translation of certain senolytics to human clinical trials.

D+Q was the first senolytic intervention to reach human clinical studies (ClinicalTrials.gov NCT02874989) only one year after the demonstration of their senolytic activity (Table 1). This open-label human pilot study examined the therapeutic effect of intermittent, oral dosing of D+Q for 3 weeks to alleviate mild to severe idiopathic pulmonary fibrosis (IPF) (175). This study demonstrated that short-term, periodic treatment with D+Q can alleviate physical dysfunction in IPF patients, showing significant improvements in 6-minute walking distance, gait speed, and repeated chair-stand times. However, this preliminary study did not fully demonstrate the clearance of SnCs by senolytics in humans. Another clinical study (NCT02848131) examining intermittent treatment with D+Q in patients with diabetic kidney disease (DKD) was the first to demonstrate that senolytics indeed decrease SnC burden in humans. Treating DKD patients with a 3-day oral course of D+Q resulted in a reduction of SA-β-gal–positive and p16^INK4a^- and p21^CIP1^-expressing cells in adipose and skin biopsies. Further, examination of patient blood samples also found a reduction in SASP factors (IL-1α, IL-6, MMP-9/12) (176). Collectively, these data demonstrated that senolytic D+Q treatment decreased SnC burden and alleviated certain SnC-associated pathologies through senolytic clearance of SnCs in humans.

The preclinical data suggested a key role of SnCs in AD. This has led to the initiation of pilot clinical trials (NCT04063124 and NCT04685590) examining the effects of intermittent D+Q treatment in adults aged 65 or above with the clinical diagnosis of early-stage AD to determine its feasibility in modulating the progression of AD and its safety profile (Table 1).

The success of senolytic treatment in alleviating multiple age-associated diseases in preclinical models as well as human pilot clinical trials triggered many clinical studies to examine the effects of D+Q, fisetin, and Unity Biotechnology’s senolytic compounds on metabolic dysfunction, frailty, AD, kidney function, osteoarthritis, COVID-19, and more (Table 1). Fisetin is currently in clinical trials to examine its therapeutic effects on kidney disease, bone health, diabetes, AD, and COVID-19. Intra-articular injection of the p53-MDM2 interaction inhibitor UBX-0101 was used to treat osteoarthritis, but the trial failed to meet its primary endpoints. The BCL-x_L_ inhibitor UBX-1325 is in a clinical trial to treat patients with diabetic macular edema or neovascular age-related macular degeneration with a single intravitreal injection. Preliminary results from this study suggested a reduction in central subfield thickness and improved visual acuity (Table 1). Another BCL-2 and BCL-X_L_ inhibitor, navitoclax, is currently being used in multiple trials for treating various cancers, including ovarian and lung cancer, in combination with other chemotherapies (NCT00445198; NCT02591095; NCT02520778; NCT02079740). Although the application of navitoclax in these trials was not intended to test the senolytic activity of navitoclax in humans, adding another arm measuring senescence markers could provide more insights into the clinical outcomes.

## Challenges and future perspectives

Given the key role of cellular senescence in driving aging and many age-related diseases, various strategies have been attempted and developed to achieve senolysis, senostasis, and even senoreversal. Indeed, the success of senotherapeutics in preclinical model systems and promising preliminary results of pilot trials have sparked numerous clinical trials to assess the effectiveness of senotherapeutic drugs in slowing disease progression, reducing disease severity, alleviating frailty, and improving resilience. However, many challenges remain.

Despite the increasing efforts to characterize SnCs and their SASP in normal physiological and specific disease models, detecting and quantifying senescence and SnCs remains challenging. Currently, there are no universal markers specific for SnCs. Increased β-galactosidase activity at pH 6.0 is widely used as a senescence marker; however, it is not a definite feature of SnCs, as it can be detected in other cells or under certain conditions (177–179). Notably, SnCs are highly heterogeneous and complex in cell types, cues, activated signaling pathways, and tissue distributions. Additionally, the cell type–specific pathophysiology in different disease contexts further contributes to difficulties in identifying bona fide senescence markers. Thus, whether a specific and universal senescence marker exists remains unclear, and multiple markers of senescence are still needed to characterize the heterogeneous SnCs. To assess the potential risk of targeting physiologically relevant SnCs, specific functional senescence markers are also needed to distinguish pathological SnCs. Alternatively, intermittent treatment of senolytics with a hit-and-run mechanism may help minimize potential side effects in clinical use.

As there are no available universal senescence markers, a pan-senolytic or pan-senotherapeutic capable of targeting all types of SnCs is unlikely to be realistic. Combining several senotherapeutic drugs and/or strategies targeting SnCs could be a way to improve efficacy and reduce side effects. For example, the combination of dasatinib and quercetin has been shown to efficiently kill a broader spectrum of SnCs than either treatment alone (88, 180). In addition, given that cellular senescence and other pillars of aging are highly interlinked, combining senotherapeutics with other types of geroprotective interventions may provide additive or synergistic therapeutic effects in aging and diseases.

The SnC surfaceome–based immunotherapies offer an alternative strategy to target SnCs by leveraging the natural immune response. However, it is challenging to identify antigens that are specifically expressed on SnCs, especially in humans. Therefore, extreme caution and extensive safety studies are required before application of these immunotherapy approaches in the clinic to treat senescence-related dysfunctions. In addition, immunotherapies are typically more expensive than small molecule–based therapies, which may limit their practical applications.

Testing senolytic efficacy in preclinical studies using animal models of natural and accelerated aging as well as chronic diseases has laid the foundation for human clinical trials (11, 58, 61, 88, 145–148, 156, 165, 170, 181, 182). Nonetheless, questions remain regarding disease stages, safety profiling, tolerability, and side effects. A primary challenge in senotherapeutics revolves around the technical difficulties in assessing the abundance of SnCs in humans. This creates further complications in determining reliable markers for an intervention study, especially when trying to dissect the specificity of senolytics in targeting and eliminating a subset of SnCs. Moreover, reduction of SnC burden alone is not sufficient to prove senolysis, as some senolytics could indirectly stimulate immune cell–mediated clearance of SnCs, further confounding the confirmation of senolysis (81, 170, 183). Also, suppression of the SASP by senomorphics can prevent the spread of secondary senescence. Therefore, in vivo senolysis should be evaluated using senescence/SASP markers along with markers of apoptosis and other cell death pathways. For example, a single-cell multi-omics approach using flow cytometry, cytometry by time of flight (CyTOF), and/or mass imaging could serve to investigate which SnC types are dying, and by what pathways, upon treatment with a given senolytic.

In summary, with the advancement of novel technologies, it can be anticipated that more progress will be made in tackling the challenges of characterizing SnCs using more biologically relevant biomarkers. Improving in vivo models of diseases and aging will also unlock the full potential of senotherapeutics and allow for the translation of senotherapeutics into clinical use.

## Figures and Tables

**Table 2:**
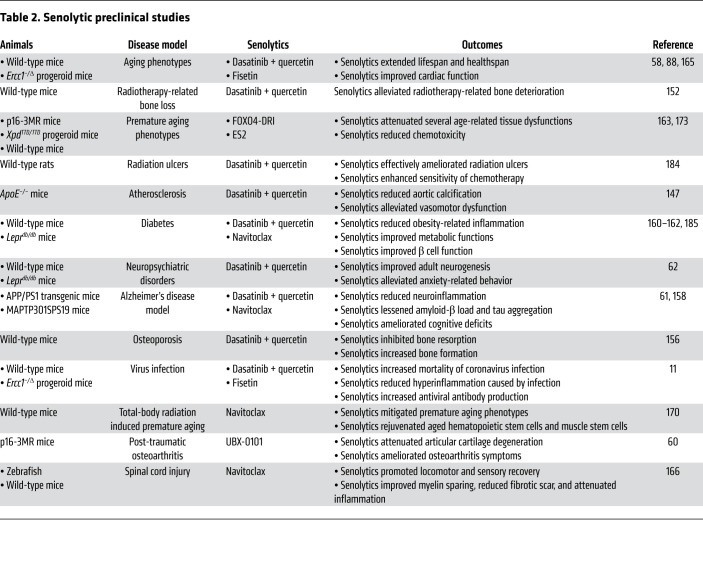
Senolytic preclinical studies

**Table 1:**
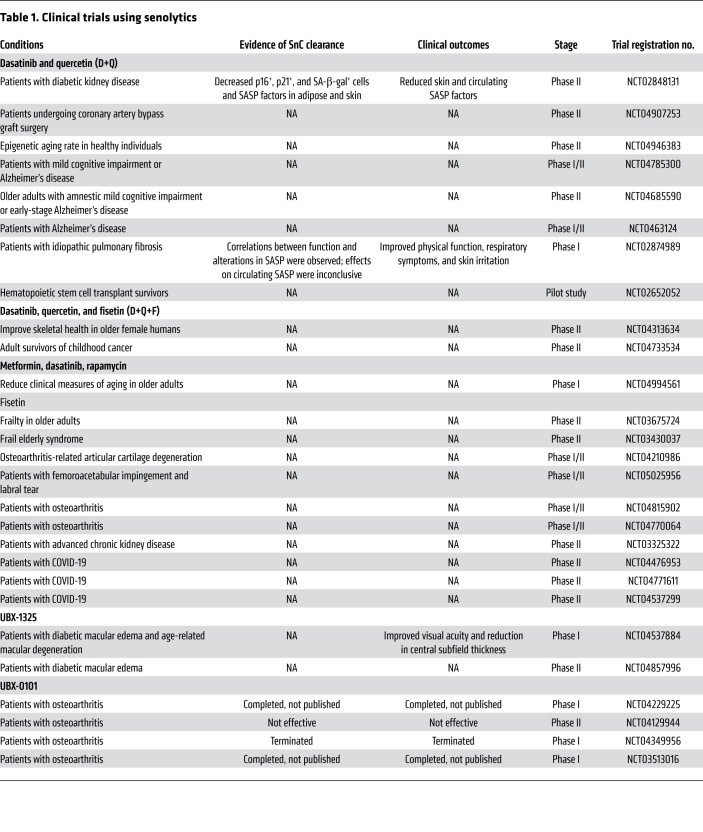
Clinical trials using senolytics

## Version 1 08/01/2022

Electronic publication
